# Primary Extracellular Matrix Enables Long-Term Cultivation of Human Tumor Oral Mucosa Models

**DOI:** 10.3389/fbioe.2020.579896

**Published:** 2020-12-04

**Authors:** Leonie Gronbach, Philipp Jurmeister, Monika Schäfer-Korting, Ulrich Keilholz, Ingeborg Tinhofer, Christian Zoschke

**Affiliations:** ^1^Institute of Pharmacy (Pharmacology and Toxicology), Freie Universität Berlin, Berlin, Germany; ^2^Institute of Pathology, Berlin Institute of Health, Humboldt-Universität zu Berlin, Corporate Member of Freie Universität Berlin, Charité – Universitätsmedizin Berlin, Berlin, Germany; ^3^Heidelberg and German Cancer Consortum Partner Site Berlin, German Cancer Research Center, Berlin, Germany; ^4^Comprehensive Cancer Center, Berlin Institute of Health, Humboldt-Universität zu Berlin, Corporate Member of Freie Universität Berlin, Charité – Universitätsmedizin Berlin, Berlin, Germany; ^5^Department of Radiooncology and Radiotherapy, Berlin Institute of Health, Humboldt-Universität zu Berlin, Corporate Member of Freie Universität Berlin, Charité – Universitätsmedizin Berlin, Berlin, Germany

**Keywords:** extracellular matrix, head and neck cancer, oral mucosa, personalized medicine, tissue engineering, tumor microenvironment, long-term cultivation, Hyalograft 3D

## Abstract

3D tumor models clearly outperform 2D cell cultures in recapitulating tissue architecture and drug response. However, their potential in understanding treatment efficacy and resistance development should be better exploited if also long-term effects of treatment could be assessed *in vitro*. The main disadvantages of the matrices commonly used for *in vitro* culture are their limited cultivation time and the low comparability with patient-specific matrix properties. Extended cultivation periods are feasible when primary human cells produce the extracellular matrix *in situ*. Herein, we adapted the hyalograft-3D approach from reconstructed human skin to normal and tumor oral mucosa models and compared the results to bovine collagen-based models. The hyalograft models showed similar morphology and cell proliferation after 7 weeks compared to collagen-based models after 2 weeks of cultivation. Tumor thickness and VEGF expression increased in hyalograft-based tumor models, whereas expression of laminin-332, tenascin C, and hypoxia-inducible factor 1α was lower than in collagen-based models. Taken together, the *in situ* produced extracellular matrix better confined tumor invasion in the first part of the cultivation period, with continuous tumor proliferation and increasing invasion later on. This proof-of-concept study showed the successful transfer of the hyalograft approach to tumor oral mucosa models and lays the foundation for the assessment of long-term drug treatment effects. Moreover, the use of an animal-derived extracellular matrix is avoided.

## Introduction

Stromal, endothelial, and immune cells create a unique environment for each individual tumor with altered paracrine signaling compared to the normal tissue (Zheng and Gao, 2019). This cellular tumor microenvironment can promote tumor growth, invasion, and dissemination (Varol, 2019) as well as treatment resistance (Jo et al., 2018). The impact of the extracellular matrix (ECM) as the major component of the tumor microenvironment in these biological processes remains contradictory or unexplored (Pickup et al., 2014; Saggioro et al., 2020). Commonly, tumors dysregulate the composition and structure of the surrounding normal tissue toward an inflamed, hypoxic, and desmoplastic tumor microenvironment (Zheng and Gao, 2019). The effect of the tumor environment on the biology of tumors of the oral cavity remains to be investigated.

Patient-specific tumor ECMs are rarely recapitulated *ex vivo*. Tumor cells are either cultivated in scaffold-free ultra-low attachment plates or embedded in collagen of animal origin, e.g., Matrigel (Langhans, 2018). Furthermore, non-human matrices like cellulose are used as scaffolds for *ex vivo* tumor models (Nath and Devi, 2016). Major drawbacks of these approaches include poor stability, limited lifespan, and underrepresentation of patient-specific tumor microenvironment components. Initially designed to better reconstruct human skin, the hyalograft-3D is a biodegradable, non-immunogenic scaffold, which consists of esterified hyaluronic acid fibers. It is certified for medical use and allows the fibroblasts to produce and assemble their own ECM (Campoccia et al., 1998). Thereby, hyalograft-based skin models extended the life by six times, compared to collagen-based skin models (Stark et al., 2006).

Recently, we developed normal and tumor oral mucosa models emulating head and neck cancer, with a collagen scaffold (Gronbach et al., 2020) to improve non-clinical drug evaluation. The 3D model showed large similarities in morphology, grading, and protein expression profiles to patient’s tumors. Moreover, the tumor models recapitulated docetaxel and cetuximab effects in line with clinical observations of head and neck-cancer. However, the cultivation of the collagen-based tumor models for a maximum of 2 weeks enabled only the investigation of short-term drug effects. This represents a major limitation for studies investigating the impact of genetic heterogeneity and therapy-driven clonal evolution in acquired drug resistance in the tumor (Magdeldin et al., 2014; Braig et al., 2017).

Herein, we assessed whether by using the hyalograft-3D approach human tumor oral mucosa models could be maintained in *ex vivo* cultures for up to 7 weeks, without major changes in tumor cell viability and proliferative activity. In addition, the impact of the ECM on tumor growth and invasion in hyalograft-based tumor oral mucosa models was compared with their collagen-based counterparts.

## Materials and Methods

### Materials

Collagen G, DMEM 10× and HEPES buffer were purchased from Merck (Darmstadt, Germany). Hyalograft-3D was purchased from Anika Therapeutics (Bedford, MA, United States). The thrombin-fibrinogen-solution tisseel^®^ was purchased from Baxter (Deerfield, IL, United States).

Human oral keratinocytes and human oral fibroblasts, as well as the respective cell culture media were purchased from ScienCell (Carlsbad, CA, United States). The tumor-cell line SCC-25 from the tongue (RRID:CVCL_1682, Rheinwald and Beckett, 1981) was a generous gift from Howard Green, Dana-Farber Cancer Institute (Boston, MA, United States). The detailed composition and origin of the construct growth and construct differentiation media were described elsewhere (Gronbach et al., 2020). Here, these media were supplemented with the transforming growth factor (TGF)-β1 and aprotinin, obtained from ThermoFisher Scientific (Waltham, MA, United States) and Merck. 12-well plates and 12-well inserts (0.4 μm pore size) were obtained from Greiner bio-one (Leipzig, Germany).

Hematoxylin, eosin, rotihistol, and rotihistokit were purchased from Carl Roth (Karlsruhe, Germany). Periodic acid was from Sigma-Aldrich and Schiff’s reagent was obtained from Merck. Primary antibodies were purchased from abcam (Cambridge, United Kingdom): hypoxia-inducible factor 1α (1:200; RRID:AB_880418), Ki-67 (1:100; RRID:AB_302459), laminin-332 (1:500; RRID:AB_1566368), Tenascin C (1:1000; RRID:AB_2043021), vascular endothelial growth factor (1:200; RRID:AB_299738). Cytokeratin Pan Plus KL1 antibody (1:100; RRID:AB_2864507) was from Zytomed (Berlin, Germany). Anti-mouse and anti-rabbit IgGs (H + L), with F(ab’)2 Fragment (Alexa Fluor^®^ 488 and 594 Conjugate; RRIDs: AB_1904025, AB_2714182) were obtained from Cell Signaling Technology (Danvers, MA, United States). DAPI (4′,6-Diamidin-2-phenylindol) mounting medium was purchased from dianova (Hamburg, Germany). The *in situ* cell death detection kit (TUNEL assay) was purchased from Sigma-Aldrich (Munich, Germany).

### Cell Culture

Human oral keratinocytes and human oral fibroblasts (ScienCell) were cultured in oral keratinocyte and fibroblast medium, respectively, at 37°C with 5% CO_2_. The SCC-25 tumor-cell line was grown in DMEM/F-12 Ham medium, supplemented with 9% fetal calf serum, 0.9% L-glutamine, and penicillin/streptomycin. The medium was changed three times a week and the cells were passaged after reaching confluency of 80%. The cell line was tested for mycoplasm and regularly checked by single nucleotide polymorphism authentication (Multiplexion; Heidelberg, Germany). Cell culture was performed according to standard operating procedures and referred to good cell culture practice.

### Multilayered Oral Mucosa Model Building

**FIGURE 1:**
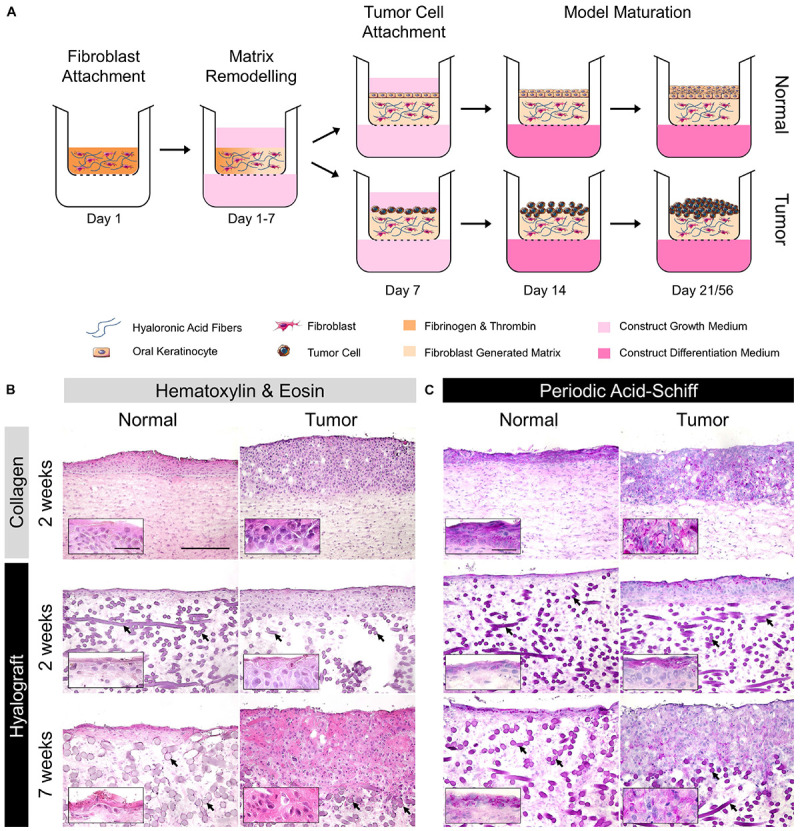
Procedure outline and morphology of NOM and TOM models. **(A)** Human oral fibroblasts were suspended in a fibrinogen/thrombin solution and poured into a patch with esterified hyaluronic acid fibers (Hyalograft-3D). Fibroblasts replaced the fibrin gel by their own extracellular matrix (day 1–7). Normal oral keratinocytes or tumor cells were seeded on day 7 onto the matrix and grew until day 21 or 56 (2 or 7 weeks with tumor cells). **(B)** Hematoxylin and eosin and **(C)** Periodic Acid-Schiff staining. Dark purple structures in both stainings of the lamina propria were hyaluronic acid fibers of the scaffold (black arrows). The inserts show the difference between normal and tumor cell morphology by higher magnification. Representative images from the analysis of up to three batches are presented. Scale bars = 250 and 50 μm in the inserts.

The multi-layered oral mucosa models (Figure 1A) were constructed as a lamina propria growing underneath an epithelium. All cultures were kept at 37°C and 5% CO_2_ in a humidified atmosphere. The building of collagen-based oral mucosa models was described previously (Gronbach et al., 2020). Briefly, 1 × 10^5^ human oral fibroblasts per model were mixed with a buffered solution and added to collagen. After solidification of the matrix, construct growth medium was added to the model and changed three times until day 7. Thereafter, either 1 × 10^6^ human oral keratinocytes or 1 × 10^6^ SCC-25 cells were seeded onto the lamina propria compartment for normal or tumor oral mucosa models, respectively. From day 14, the construct surface was kept medium-free to expose the epithelium to the air and the construct growth medium was supplemented with 0.25 mmol/l ascorbic acid acting as construct differentiation medium. On day 21, the models were snap frozen and stored at −80°C.

The generation of hyalograft-3D was described previously (Stark et al., 2006). In brief, the hyalograft-3D is a fleece-like matrix, composed of recombinant human hyaluronic acid fibers, esterified with benzylic alcohol to retard its degradation. Here, hyalograft-3D was cut into disks of 10 mm in diameter to fit the size of 12-well cell culture inserts. Next, 1 × 10^5^ human oral fibroblasts per model were resuspended in a thrombin solution (10 international units/ml), mixed with a fibrinogen solution (8 mg/ml) and subsequently added to the pre-cut hyalograft-3D pieces. During the following 7 days, the fibroblasts were allowed to replace the fibrin by *in situ* produced ECM components (Stark et al., 2006). Thereafter, either human oral keratinocytes or SCC-25 cells were seeded onto the lamina propria compartment as described above for the collagen model. The construct growth medium was supplemented with 1 ng/ml transforming growth factor-β1 and 500 international units/ml aprotinin. TGF-β1 reduces keratinocyte differentiation and growth (Dahler et al., 2001). Aprotinin, a serin-protease inhibitor was used to limit fibrinolysis and thus premature model degradation. Medium was changed three times per week. From day 14, the construct surface was kept medium-free and aprotinin was reduced to 200 international units/ml in the construct differentiation medium. At the end of the cultivation period, the models were snap frozen and stored at −80°C.

### Morphology and Protein Expression

The models were cut into 7 μm thick slices using a cryotome (Leica CM 1510S; Leica, Wetzlar, Germany) and fixed with 4% paraformaldehyde. The cryosections were subjected to either hematoxylin and eosin (H&E), periodic acid-Schiff (PAS), immunofluorescence staining or immunohistochemistry (IHC). For the H&E staining, slides were successively submerged into hematoxylin (5 min), water (5 min), eosin (30 s), 70 and 99.9% ethanol (2 min) and rotihistol (2 min). Finally, the slides were fixed with the rotihistokit and a cover slide. PAS staining was performed on a Tissue-Tek Prisma Plus Automated Slide Stainer (Sakura Finetech, Staufen, Germany). Slides were incubated with periodic acid for 10 min, followed by staining with Schiff’s reagent for 10 min and hematoxylin for 7 min. For immunofluorescence staining, the samples were permeabilized for 5 min by a 0.5% triton solution, blocked for 30 min with 5% goat serum and incubated over night with the primary antibody at 4°C. Afterward the slides were incubated for 1 h with the secondary antibody. In the end, DAPI mounting medium was added to stain cell nuclei and fixed the samples. IHC staining was done on a BOND MAX Automated Slide Stainer (Leica) using the HP1 program and the BOND polymer Refine Detection System (Leica). Images were taken with a fluorescence microscope (BZ-8000; Keyence, Neu-Isenburg, Germany) and analyzed using the ImageJ software (Schneider et al., 2012).

### Apoptosis Quantification

For apoptosis measurements, the *in situ* cell death detection kit was used according to the manufacturer’s instructions. The kit detects DNA fragments in apoptotic cells based on TdT-mediated dUTP-biotin nick end labeling (TUNEL).

### Data Analysis

Data are presented as the mean + standard deviation (SD) obtained from up to three independent experiments. Due to the explorative data analysis, a level of *p* ≤ 0.05, calculated using non-parametric Kruskal–Wallis tests and subsequent Dunn’s *Post hoc*-tests, was considered to indicate a statistically significant difference.

## Results

### Morphological Analysis

We extended the culture period from 2 weeks of collagen-based normal oral mucosa models (c-NOM) and tumor oral mucosa models (c-TOM) to 7 weeks in hyalograft-based h-NOM and h-TOM models. To evaluate the impact of the scaffold, we cultured also h-NOM and h-TOM for 2 weeks (Figure 1A).

The epithelium of c-NOM models consisted of a basal layer with rounded cells and multiple layers of spinous cells, as found in non-keratinized oral mucosa (Figure 1B). All TOM models depicted an unstructured, hyperproliferative, and thickened epithelial layer with atypical, enlarged, irregular tumor cells and hyperchromatic nuclei. The tumor morphology appeared desmoplastic in particular in h-TOM models after 7 weeks of culture (Figure 1B, inserts).

**FIGURE 2:**
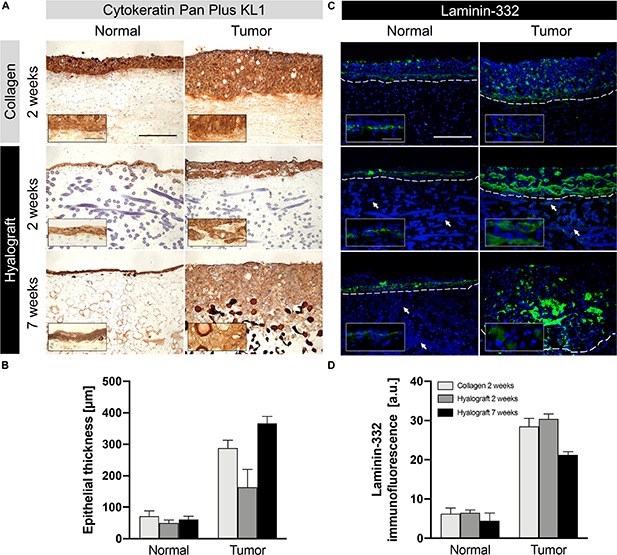
Expression of cytokeratin and laminin-332 in NOM and TOM models. **(A)** Cytokeratin staining (brown) showed the absence of invasive growth in NOM and c-TOM models. In contrast, nests of cytokeratin positive cells started to infiltrate the lamina propria in h-TOM models after 2 weeks and markedly separated into single cells after 7 weeks. **(B)** Epithelial thickness in TOM models exceeded those in NOM models. The highest value occurred in h-TOM models after 7 weeks. **(C,D)** Laminin-332 (green) expression was restricted to a small layer in NOM models and diffusely clustered in TOM models. DAPI stained nuclei and fibers in blue, which could however be distinguished by their size and shape. The inserts show the border between epithelial cells and the matrix, with highest infiltration of the tumor cells in the 7 weeks cultured hyalograft-models, by higher magnification. White arrows highlight fibers and dashed lines indicate the border between epithelium and lamina propria. Representative images from up to three independent cultures are presented. Scale bars = 250 and 50 μm in the inserts. Bar graphs show the mean + SD from the quantitative analysis of up to six regions of interest.

The glycogen distribution was confined to the upper epithelial layers of the h-NOM model, while glycogen was found in all epithelial layers of c-NOM models (Figure 1C). A similar pattern was observed in TOM models after 2 weeks of culture (Figure 1C, inserts). Only after 7 weeks of culture the glycogen distribution became also patchy in h-TOM models. Concurrently, cytokeratin-positive tumor cells penetrated the hyalograft-3D matrix only slightly as tumor nests, but massively invaded the lamina propria compartment as single cells (Figure 2A). The final tumor thickness in h-TOM models exceeded tumor thickness of c-TOM models, but the difference was not statistically significant (Figure 2B).

The large structures in the lamina propria of hyalograft-based models were hyaluronic ester fibers, which were unspecifically stained by hematoxylin and eosin, periodic acid-Schiff as well as DAPI. The unspecific staining might be explained by the large three-dimensional structure of the fibers and their negative charge, which prevented the washout of stains as well as monoclonal antibodies, and led to the intercalation of DAPI into the fibers.

### Protein Expression

The basement membrane protein laminin-332 was expressed in particular between the epithelial layer and the lamina propria in both h-NOM and c-NOM models (Figure 2C). In contrast, the expression of laminin-332 was more heterogeneous in TOM models with the highest levels in h-TOM models after 2 weeks of culture (Figure 2D), in particular observed in the subepithelial zone in h-TOM models.

**FIGURE 3:**
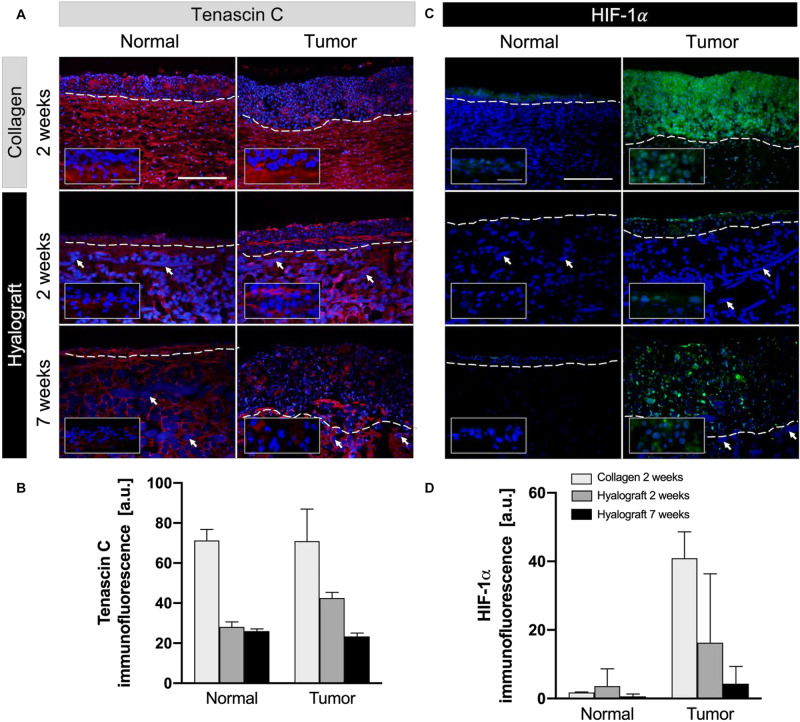
Expression of tenascin c and HIF-1α in NOM and TOM models. **(A,B)** Tenascin c (red) was less expressed in hyalograft-based models than collagen-based models and further decreased during cultivation. **(C,D)** HIF-1α (green) showed increased expression in TOM models, with matrix-dependent differences. DAPI stained nuclei and fibers in blue. The inserts show the border between epithelial cells and the matrix for the tenascin C staining and highlights of the HIF-1α staining by higher magnification. White arrows highlight fibers and dashed lines indicate the border between epithelium and lamina propria. Representative images from up to three independent cultures are presented. Scale bar = 250 and 50 μm in the inserts. Bar graphs show the mean + SD from the quantitative analysis of up to six regions of interest of interest.

The extracellular matrix protein tenascin C was most abundant in collagen-based models with no difference between NOM and TOM models (Figure 3A). Tenascin C expression markedly decreased in hyalograft-based models already after 2 weeks of cultivation and further declined to 33% (*p* > 0.05) after 7 weeks. Again, no relevant difference between NOM and TOM models was observed (Figure 3B).

The hypoxia-inducible factor (HIF)-1α was detected in the entire tumor mass of c-TOM models, and particularly in central tumor areas in h-TOM models (Figure 3C). Very low levels of HIF-1α were detected in both c-NOM and h-NOM models (Figure 3D).

**FIGURE 4:**
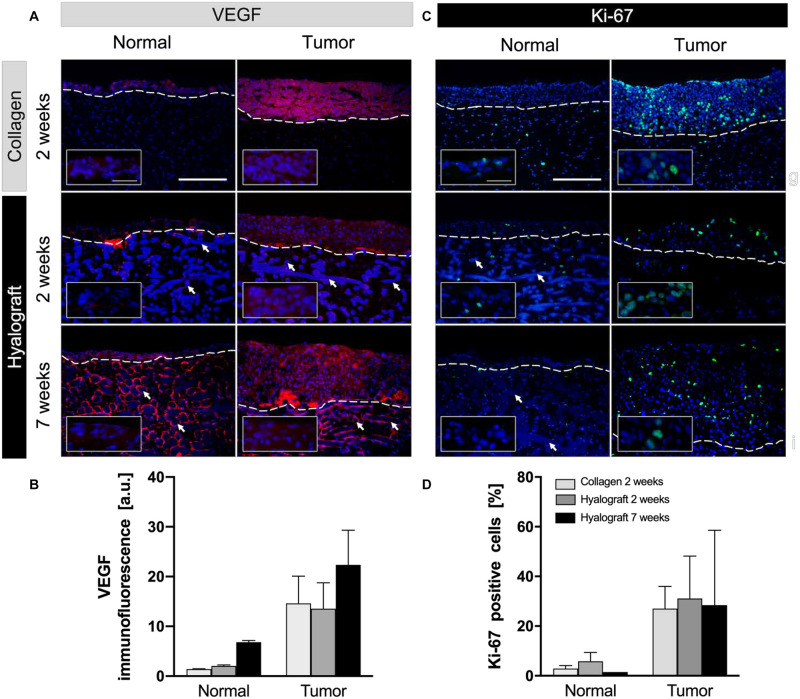
Expression of vascular endothelial growth factor (VEGF) and proliferation (Ki-67) in NOM and TOM models. **(A,B)** Highest expression of VEGF (red) was observed in h-TOM models after 7 weeks. **(C,D)** The number of proliferative cells (Ki-67 positive, green) was increased in TOM models until the end of the cultivation period compared to NOM models. The inserts highlight detected VEGF and Ki-67 in the epithelial layers by higher magnification. Representative images from up to three independent cultures are presented. Scale bars = 250 and 50 μm in the inserts. Bar graphs show the mean + SD from the quantitative analysis of up to six regions of interest.

Overall, vascular endothelial growth factor (VEGF) was expressed at similar levels in the c-TOM and h-TOM models; however, the type of matrix interfered with its localization. While VEGF was detected in the entire tumor areas of c-TOM models, it was restricted to the border between the tumor layer and the lamina propria in h-TOM models (Figures 4A,B). VEGF expression further increased after 7 weeks both in NOM and TOM models (*p* > 0.05). Increased VEGF levels were particularly observed close to hyaluronic acid fibers in h-NOM models.

### Proliferation and Apoptosis

Proliferation was higher in tumor compared to normal models, irrespective of the used matrix (Figures 4C,D). Importantly, tumor cells continued to proliferate excessively in h-TOM models until the end of the 7-week culture within all regions of the culture.

In c-NOM and the 7 weeks cultured h-NOM models, only few apoptotic cells could be detected (mean = 2.5%), while in the 2 weeks h-NOM models apoptotic cells made up to 20% of the epithelial cells (Supplementary Figure 1).

In TOM models, both 2 weeks cultured c-TOM and h-TOM models depicted less than 5% apoptotic cells, but the 7 weeks cultured h-TOM models showed up to 30% apoptotic cells.

## Discussion

We here showed that normal and tumor oral mucosa models can be successfully cultured in a hyalograft-based scaffold, allowing extended *ex vivo* cultivation. Our data corroborate previous findings showing that hyaluronic acid and its derivatives provide a well-defined and tunable scaffold for *ex vivo* tumor models (Fong et al., 2014). Moreover, hyalograft-based models are not affected by the poor adhesion of epithelial layers and the tendency to shrink of collagen-based models (Stark et al., 2004). In contrast, nylon-meshes and collagen-chitosan-sponges, which have been tested for elongated cultivation periods have the disadvantage of requiring long pre-cultivation and displaying considerable stiffness, thus complicating tissue sectioning and analysis (Michel et al., 1999; Stark et al., 2006).

Hyalograft-based tumor models contained high numbers of proliferative cells and recapitulated hallmarks of oral cancer even after a cultivation period of 7 weeks. In particular, increased epithelial thickness, abundant cellular pleomorphism, and the altered laminin-332 expression, very well reflected the histopathological characteristics of patient tumors (Miyazaki, 2006; Bernstein et al., 2013; Jerjes et al., 2019). Thus, h-TOM models should be suitable to monitor long-term tumor progression as well as the effects of anti-proliferative drugs and the potential tumor re-growth after an initial treatment cycle. An improved understanding of the re-growth kinetics after drug treatment would help to overcome drug resistance, which is currently the major cause of treatment failure (Vasan et al., 2019).

Beside large similarities in protein expression patterns of hyalograft-and collagen-based models, there was a significant difference in tenascin C expression. The increased expression of tenascin C in collagen-based models might explain the faster growth in the epithelial layers of both c-NOM and c-TOM models, since tenascin C is known as a provisional matrix for keratinocyte growth (Pellegrini et al., 1999). Moreover, the expression of the extracellular matrix proteins tenascin c and fibronectin discriminates low- and high-risk tongue cancers (Sundquist et al., 2017). Low tenascin C expression in the h-TOM model established from SCC-25 cells is in line with the previously described poorly invasive phenotype of this cell line model (Ramos et al., 1997).

Normal oral fibroblasts better confined tumor invasion in hyalograft- than in collagen-based models after 2 weeks. This difference might be related to paracrine signaling between fibroblasts and tumor cells which has been shown to depend on the composition of the ECM (Barcellos-Hoff and Bissell, 1989; Boudreau and Bissell, 1998). Laminin-332 appears to play a key part in the invasion process, in line with its higher expression in h-TOM compared to c-TOM models. While a well-defined laminin-332 expression is typical for normal tissues, clustered laminin-332 expression is known to promote cell survival and tumorigenesis, especially in squamous cell carcinoma (Marinkovich, 2007). In addition, the occurrence of desmoplasia in h-TOM models might contribute to delayed invasive growth and reduced hypoxia compared to c-TOM models. These differences in the ECM of collagen- and hyalograft-based models need to be considered in evaluating drug effects since hypoxia reduces the clinical efficacy of anticancer drugs (Brennan et al., 2005; Johnstone and Logan, 2006).

Although this proof-of-concept study shows the suitability of the hyalograft scaffold for the *ex vivo* cultivation of TOM models, future studies need to elucidate the scaffold effects on patient-derived tumor cells and compare these results to *in vivo* tumors. One limitation of the current h-TOM model is the relative high percentage of apoptotic tumor cells in long-term cultures. Further approaches for model improvement in the future might thus include also testing of additional supplements to the construct growth medium. Moreover, future studies will show whether the hyalograft approach better recapitulates the interaction of immune and tumor cells in an immunocompetent model of oral mucosa tumor, which seems very likely since the scaffold is non-immunogenic (Galassi et al., 2000). Given their close correlation to the individual tumor, long-term cultivation of human TOM models offer the opportunity to study tumor re-growth and alterations in the tumor stroma after initial treatment and thus will help to better understand drug resistance mechanisms.

## Conclusion

The hyalograft-3D approach recapitulated key features of human oral squamous cell carcinoma in multi-layered *ex vivo* tumor models for up to 7 weeks. The long-term cultivation provides the basis for studying tumor re-growth and stromal alterations following an initial anti-cancer drug therapy. Moreover, the well-defined and tunable hyaluronic acid derivatives might help to better culture patient-derived cells. Finally, hyalograft-based models can be extended by the addition of further tumor stroma components and relinquish the use of animal-based scaffolds.

## Data Availability Statement

The raw data supporting the conclusions of this article will be made available by the authors on reasonable request.

## Author Contributions

LG and CZ: conceptualization and design. LG, PJ, and CZ: investigation. CZ: project administration. MS-K, UK, and CZ: supervision. LG and CZ: visualization. LG and CZ: writing-original draft preparation. LG, MS-K, IT, and CZ: writing-review and editing. All authors contributed to the article and approved the submitted version.

## Conflict of Interest

The authors declare that the research was conducted in the absence of any commercial or financial relationships that could be construed as a potential conflict of interest.
