# Supplementary material for: Primary Extracellular Matrix Enables Long-Term Cultivation of Human Tumor Oral Mucosa Models
Source: Front Bioeng Biotechnol. 2020 Dec 4;8:579896. doi: 10.3389/fbioe.2020.579896 (PMC7746540; doi:10.3389/fbioe.2020.579896)
Supplement: Supplementary file 1 [file Data_Sheet_1.PDF]

Primary Extracellular Matrix Enables Long-Term Cultivation of Human Tumor Oral Mucosa Models

Leonie Gronbach*[1], *Philipp Jurmeister*[2,3], *Monika Schäfer-Korting*[1], *Ulrich Keilholz*[4], *Ingeborg Tinhofer*[3,5], *Christian Zoschke*[1]*

[1]Freie Universität Berlin, Institute of Pharmacy (Pharmacology & Toxicology), Königin-Luise-Str. 2+4, 14195 Berlin, Germany

[2]Charité – Universitätsmedizin Berlin, corporate member of Freie Universität Berlin, Humboldt-Universität zu Berlin, and Berlin Institute of Health, Institute of Pathology, Charitéplatz 1, 10117 Berlin, Germany

[3]German Cancer Research Center (DKFZ), Heidelberg and German Cancer Consortum (DKTK) Partner Site Berlin, Berlin, Germany

[4]Charité – Universitätsmedizin Berlin, corporate member of Freie Universität Berlin, Humboldt-Universität zu Berlin, and Berlin Institute of Health, Comprehensive Cancer Center, Charitéplatz 1, 10117 Berlin, Germany

[5]Charité – Universitätsmedizin Berlin, corporate member of Freie Universität Berlin, Humboldt Universität zu Berlin, and Berlin Institute of Health, Department of Radiooncology and Radiotherapy, Charitéplatz 1, 10117 Berlin, Germany

*** Correspondence:**
Christian Zoschke
christian.zoschke@fu-berlin.de

Keywords:
extracellular matrix, head and neck cancer, oral mucosa, personalized medicine, tissue engineering, tumor microenvironment, long-term cultivation, Hyalograft 3D

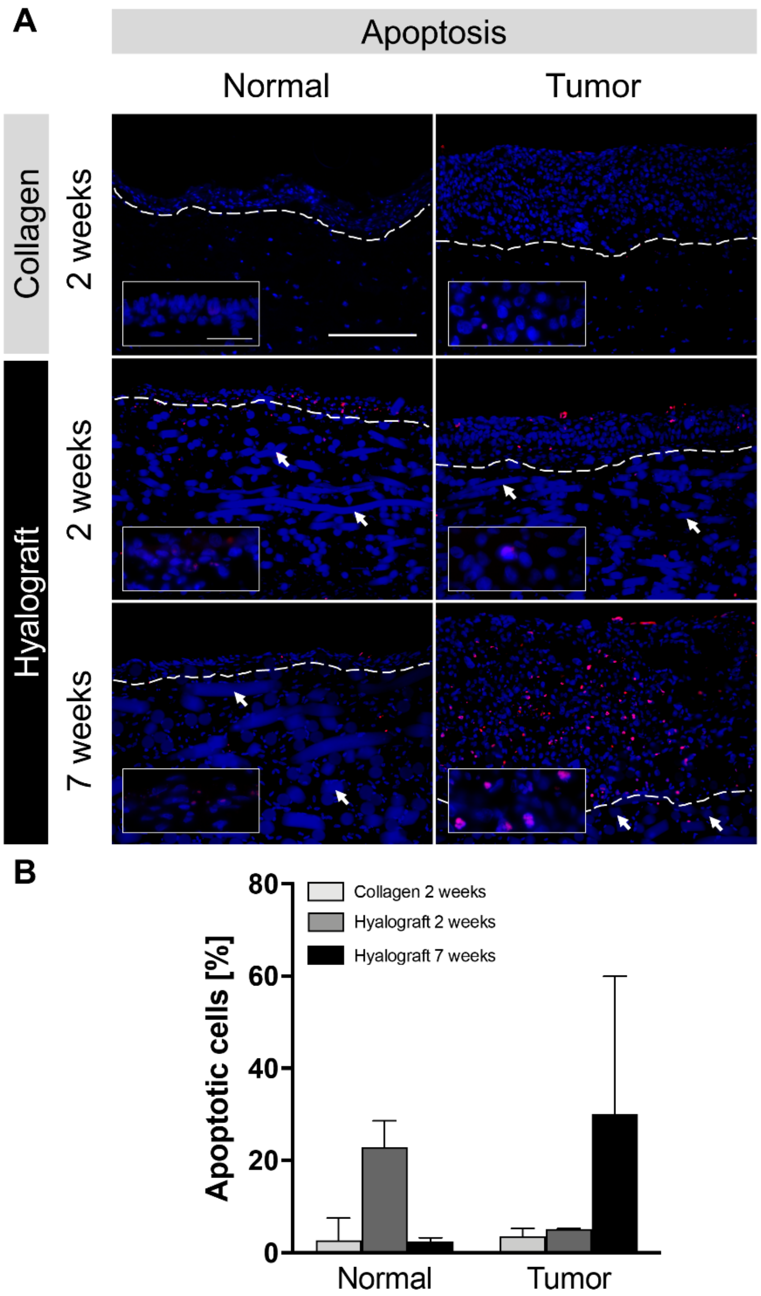

Supplementary Figure 1 | Apoptosis in NOM and TOM models. **(A)** Localization of apoptotic cells (red) by TUNEL assay and **(B)** their quantification. The percentage of apoptotic cells peaked in h-NOM models after two weeks cultivation period and in h-TOM models after seven weeks of culture. DAPI stained nuclei and fibers in blue, which could however be distinguished by their size and shape. The inserts highlight detected apoptotic cells in the epithelial layers by higher magnification. White arrows highlight fibers and dashed lines indicate the border between epithelium and lamina propria. Representative images from up to three independent cultures are presented. Scale bar = 250 µm and 50 µm in the inserts. Bar graphs show the mean + SD from the quantitative analysis of up to six regions of interest.
